# The Effectiveness of Electronic Screening and Brief Intervention for Reducing Levels of Alcohol Consumption: A Systematic Review and Meta-Analysis

**DOI:** 10.2196/jmir.3193

**Published:** 2014-06-02

**Authors:** Kim Donoghue, Robert Patton, Thomas Phillips, Paolo Deluca, Colin Drummond

**Affiliations:** ^1^Institute of PsychiatryAddictions DepartmentKing's College LondonLondonUnited Kingdom

**Keywords:** alcohol drinking, intervention studies, Internet, computers, meta-analysis

## Abstract

**Background:**

Electronic screening and brief intervention (eSBI) has been shown to reduce alcohol consumption, but its effectiveness over time has not been subject to meta-analysis.

**Objective:**

The current study aims to conduct a systematic review and meta-analysis of the available literature to determine the effectiveness of eSBI over time in nontreatment-seeking hazardous/harmful drinkers.

**Methods:**

A systematic review and meta-analysis of relevant studies identified through searching the electronic databases PsychINFO, Medline, and EMBASE in May 2013. Two members of the study team independently screened studies for inclusion criteria and extracted data. Studies reporting data that could be transformed into grams of ethanol per week were included in the meta-analysis. The mean difference in grams of ethanol per week between eSBI and control groups was weighted using the random-effects method based on the inverse-variance approach to control for differences in sample size between studies.

**Results:**

There was a statistically significant mean difference in grams of ethanol consumed per week between those receiving an eSBI versus controls at up to 3 months (mean difference –32.74, 95% CI –56.80 to –8.68, *z*=2.67, *P*=.01), 3 to less than 6 months (mean difference –17.33, 95% CI –31.82 to –2.84, *z*=2.34, *P*=.02), and from 6 months to less than 12 months follow-up (mean difference –14.91, 95% CI –25.56 to –4.26, *z*=2.74, *P*=.01). No statistically significant difference was found at a follow-up period of 12 months or greater (mean difference –7.46, 95% CI –25.34 to 10.43, *z*=0.82, *P*=.41).

**Conclusions:**

A significant reduction in weekly alcohol consumption between intervention and control conditions was demonstrated between 3 months and less than 12 months follow-up indicating eSBI is an effective intervention.

## Introduction

The hazardous and harmful use of alcohol is a global problem, contributing 4.6% of the total global burden of disease, with the highest rates reported in the European and American regions (17.3% and 14.2%, respectively) [1]. It is well documented that those with problem alcohol use seldom seek help [2]; this may be due to problems accessing treatment, or an unwillingness to do so, or failure of clinicians to identify their problem [3]. There is a large body of research to support the effectiveness of opportunistic screening and brief intervention (SBI) in reducing alcohol consumption and other alcohol-related outcomes in a number of health care settings, including primary care [4,5] and the emergency department [6,7]. A brief intervention typically comprises a single face-to-face session, ranging from 5-30 minutes in duration, and up to a maximum of 4 sessions aimed at providing information and advice that is designed to achieve a reduction in hazardous/harmful alcohol consumption [4]. Despite the effectiveness of SBI, there are a number of barriers to its widespread implementation in health care settings. Health care staff report that they lack the time and resources to carry out training and delivery of SBI in routine practice and that they lack the skills and knowledge necessary to do so [8,9].

The widespread use of computers, the Internet, and smartphones has led to the development of electronic systems to deliver SBI that can potentially address some of the barriers to implementation of traditional face-to-face SBI. Electronic SBI (eSBI) has the potential to offer greater flexibility and anonymity for the individual and reach a larger proportion of the in-need population. For both adults and adolescents, eSBI (computer-, Web-, and phone-based) can offer effective delivery of interventions in both educational and health care settings that may prove to be more acceptable than more traditional (face-to-face) approaches [10-12]. Also, eSBI can offer a more cost-effective alternative to face-to-face interventions. Previous studies have shown that 1 in 8 individuals respond to SBI; therefore, large numbers of people need to be screened to obtain a time-limited effect in reduction in alcohol consumption [4,5]. With the advent of mobile and e-technologies potentially increasing the population coverage of SBI, the potential cost of delivery can be reduced because the main cost is incurred during development of the intervention with limited additional costs associated with its delivery [13]. Evidence from recent systematic reviews has found eSBIs to be effective in reducing alcohol consumption [14,15]. However, these reviews did not address the effect of length of follow-up on alcohol outcomes. Cunningham and colleagues [16,17] conducted a randomized controlled trial of the effectiveness of an Internet-based intervention for alcohol misuse. They found that at 3- and 6-month follow-ups, those who had received the intervention had a greater reduction in alcohol consumption compared to controls. However, at 12-month follow-up the beneficial effects of the intervention were no longer apparent.

The current study aims to conduct a systematic review and meta-analysis of the available literature to determine the effectiveness of eSBI over time in nontreatment-seeking hazardous/harmful drinkers.

## Methods

### Search Strategy

A systematic search of the literature was conducted to identify randomized controlled trials investigating the effectiveness of eSBI to reduce alcohol consumption. Relevant studies were identified through searching the electronic databases PsychINFO, Medline, and EMBASE in May 2013. The search strategy was adapted from the search terms used for the National Institute for Health and Care Excellence (NICE) guideline systematic review for the effectiveness of acamprosate/naltrexone [18], and the search terms used for the Cochrane systematic review for the effectiveness of SBI for alcohol misuse [4], combined with additional search terms specific to electronic interventions to ensure a comprehensive search of the available published literature. The search terms used for this review are listed in Table 1. No date or language restrictions were applied. In addition, the reference lists of relevant review articles and key papers were hand searched. Unpublished literature was considered to be beyond the scope of this review.

**Table 1:** Electronic database search terms.

Search term topic	Search terms
**Terms for alcohol use**	
	1. alcohol-related disorder.mp.
	2. alcohol drinking.mp.
	3. (alcohol and (use$ or abuse or misuse or dependen$ or drink$ or intoxication$ or disorder$ or consumption)).mp.
	4. exp Alcoholism/ or (alcoholi$).mp.
	5. ((hazard$ or binge or heavy or harmful or risk$) and drink$).mp.
	6. 1 or 2 or 3 or 4 or 5
	7. limit 6 to abstracts
	8. (drinker$1 or (drink$ adj2 use$1) or ((alcohol$ or drink$) adj5 (binge$ or disorder$ or harm$ or hazard$ or heavy or high risk or intoxicat$ or misus$ or problem$))). ti.ab.
	9. 7 or 8
**Terms for e-formats**	
	10. exp Text Messaging/ or ((text-messag$) or (SMS) or (short message service) or (text adj messag$)).mp.
	11. ((phone adj application$) or (phone adj app)).ti,ab,kw.
	12. ((social-network) or ( social network) or (social-media) or (social-media)).ti,ab,kw.
	13. skype.ti,ab,kw.
	14. exp telemedicine/
	15. facebook.ti,ab,kw.
	16. ((personal adj digital adj assistant) or pda).ti,ab,kw.
	17. (surf$ near4 internet$).ti,ab,kw.
	18. (surf$ near4 web$).ti,ab,kw.
	19. (virtual adj reality).ti,ab,kw.
	20. Second life.ti,ab,kw.
	21. User-computer interface/
	22. (consumer adj health adj informatics$).ti,ab,kw.
	23. ((e adj health) or e-health or (electronic adj health)).ti,ab,kw.
	24. (interactive adj ((health adj communicat$) or televise$ or video$ or technolog$ or multimedia)).ti,ab,kw.
	25. ((bulletin adj board$) or bulletinboard$ or messageboard$ or (message adj board$)).ti,ab,kw.
	26. (blog$ or web-log$ or weblog$ ).ti,ab,kw.
	27. ((chat adj room$) or chatroom$).ti,ab,kw.
	28. (online or on-line).ti,ab,kw.
	29. exp internet/ or ((internet adj based) or internet-based).ti,ab,kw.
	30. ((web adj based) or web-based).ti,ab,kw.
	31. ((world adj wide adj web) or (world-wide-web) or WWW or (world-wide adj web) or (worldwide adj web) or website$).ti,ab,kw.
	32. ((electronic adj mail) or email$ or email$).ti,ab,kw.
	33. (((mobile or cellular or cell or smart) adj (phone$ or telephone$)) or smartphone).ti,ab,kw.
	34. ((CD adj ROM) or cd-rom or cdrom or (compact adj dis$)).ti,ab,kw.
	35. (decision adj (tree$ or aid$)).ti,ab,kw.
	36. (Internet or (local adj area adj network)).ti,ab,kw.
	37. (computer$ or microcomputer$ or laptop).ti,ab,kw.
	38. exp Software-/
	39. exp Computer-Graphics/
	40. exp Public-Health-Informatics/
	41. exp Audiovisual-Aids/
	42. exp Decision-Support-Techniques/
	43. exp Medical Informatics/
	44. exp Computer-Systems/
	45. (or/10-44)
**Brief interventions**	
	46. alcohol reduction.mp.
	47. brief intervention.mp.
	48. early intervention.mp.
	49. minimal intervention.mp.
	50. alcohol therapy.mp.
	51. Harm Reduction/
	52. screening.mp.
	53. (counseling or counselling).mp.
	54. controlled drinking.mp.
	55. (brief counseling or brief counselling).mp.
	56. physician based intervention.mp.
	57. general practitioner intervention.mp.
	58. Secondary Prevention/
	59. general practitioner’s advice.mp.
	60. brief physician-delivered counseling.mp.
	61. brief nurse-delivered counseling.mp.
	62. identification.mp.
	63. intervention.mp.
	64. or/46-63
**Terms for randomized controlled trial**	
	65. exp clinical trial/ or (crossover procedure or double blind procedure or placebo$ or randomization or random sample or single blind procedure).sh.
	66. exp clinical trial/ or cross-over studies/ or double-blind method/ or random allocation/ or randomized controlled trials as topic/ or single-blind method/
	67. exp clinical trial/ or (placebo or random sampling).sh.
	68. (clinical adj2 trial$).tw.
	69. (crossover or cross over).tw.
	70. (((single$ or doubl$ or trebl$ or tripl$) adj5 blind$) or mask$ or dummy or singleblind$ or doubleblind$ or trebleblind$ or tripleblind$).tw.
	71. (placebo$ or random$).mp.
	72. (clinical trial$ or controlled clinical trial$ or random$).pt. or treatment outcome$.mp.
	73. animals/ not human$.mp.
	74. animal$/ not human$/
	75. (or/65-72) not (or/73-74)
	76. and/9,45,64,75

### Selection Criteria

The inclusion criteria for this review were as follows:

1. Randomized controlled, parallel group trial comparing eSBI with a control condition (ie, care as usual, assessment only, nonintervention);
2. Participants were identified, through screening, as consuming alcohol to a hazardous level;
3. Measured alcohol reduction by independent reports of drinking quantity (eg, average consumption of alcohol per specified time period), including self-reports or reports from others of drinking frequency (eg, number of drinking occasions per specified time period), drinking intensity (eg, number of drinks per drinking day), or drinking within recommended limits (eg, official recommendations per specified time period), or levels of laboratory markers of reduced alcohol consumption, such as serum gamma-glutamyltransferase (GGT) or mean corpuscular volume (MCV); and
4. Trial arms had at least 10 participants.

We defined eSBI as an electronic intervention aimed at providing information and advice designed to achieve a reduction in hazardous/harmful alcohol consumption with no substantial face-to-face therapeutic component. SBI was defined as a brief intervention comprised of a single session, ranging from 5-45 minutes in duration, and up to a maximum of 4 sessions aimed at providing information and advice designed to achieve a reduction in hazardous/harmful alcohol consumption. Studies were not deemed eligible for inclusion if participants were alcohol dependent, mandated to complete eSBI, or a preselected specific group such as pregnant women. There were no restrictions on age.

### Identification of Included Studies

After each search, references were downloaded to the electronic bibliographic management software EndNote and duplicates were removed. Relevant titles were first identified and then abstracts were screened against inclusion criteria. If insufficient information was available in the abstract, the full text was retrieved. Eligibility was confirmed by at least one other member of the review group. The methodological quality of each study was assessed using the Scottish Intercollegiate Guidelines Network (SIGN) validated checklist [19]. Each question in the checklist covers an aspect of research methodology and was rated as present, absent or “can’t say” if inadequate information was available in the research article. An overall rating of quality was assigned to each article based on the checklist criteria:

1. High quality: Majority of criteria met with little risk of bias and conclusions unlikely to change by further research.
2. Adequate: Most of the criteria met with some risk of bias and conclusions may change in light of further research.
3. Low quality: Most criteria not met or significant flaws relating to key aspects of the study design and conclusions likely to change in light of future research.

### Data Extraction

A Microsoft Word-based form was used to extract data from eligible research papers. Data extraction was conducted independently by 2 members of the research team and consensus agreement reached by discussion between the 2 members if discrepancies arose. An intention-to-treat analysis was used wherever possible. If the study was a 3-arm trial, the control group sample size was divided by 2; if it was a 4-arm trial, it was divided by 3 to avoid double counting.

### Data Analysis

For the continuous variable (grams ethanol consumed per week) the mean difference was weighted using the random-effects method based on the inverse-variance approach to control for differences in sample size between studies. Alcohol consumption data are often not normally distributed. Because of this, some studies reported the sample median and range/interquartile range (IQR) and not the mean and standard deviation (SD). If appropriate data were not available in the published research papers, to calculate an effect size (ie, the mean, SD, and sample size), authors were contacted to request the required data. If the authors were unable to provide this data, the mean and SD were imputed from the median and range using the method proposed by Hozo et al [20]. If only the median and the IQR were available, the median was taken as an estimate of the mean and the IQR was divided by 1.35 (the distance in SDs from the mean). If appropriate data to estimate an effect size could not be obtained or imputed, the trial was not included in the meta-analysis. Some of the studies included in the meta-analysis had more than one trial arm. The number of participants in the control arm was divided by 2 for a 3-arm trial and by 3 for a 4-arm trial to avoid double counting and undue weighting.

Alcohol consumption, reported as the number of standard drinks per week, was converted into grams of ethanol per week using the definition for a standard drink reported in the research article. If this was not reported, the established standard for the country in which the research took place was used [21]. If alcohol consumption was reported per month versus per week, it was adjusted by multiplying by 52/12, or multiplied by 7 if reported as grams per day [4].

To check for the consistency of effects across studies, Cochran *Q* was calculated to determine the presence of heterogeneity and the magnitude was measured using I^2^. The I^2^ statistic was interpreted in the following way based on Higgins et al [22]: Research studies that produce statistically significant results may be more likely to be published than those with nonstatistically significant results, resulting in a “file-drawer” effect. Similarly, those studies that produce results in an opposite direction to that hypothesized and have a small sample size may be less likely to be published. This is referred to as publication bias and it was assessed using funnel plots and Egger’s weighted regression method. A significant Egger’s test indicates the possibility of the presence of publication bias.

The length of follow-up period can vary between individual studies and there may be more than one point of follow-up per study. Therefore, subgroup analysis was performed for up to 3 months, between 3 and less than 6 months, between 6 and less than 12 months, and 12 months or greater follow-up length postintervention.

## Results

### Study Characteristics

**Figure 1:**
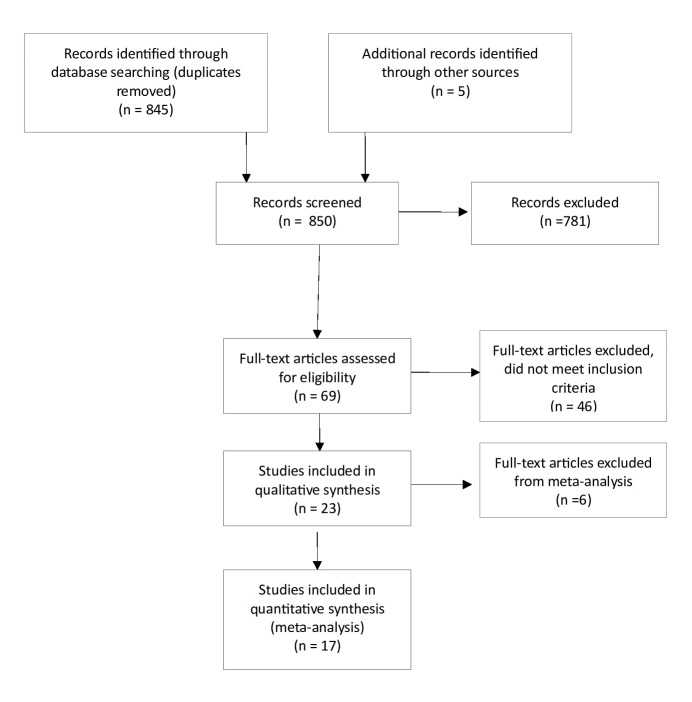
Flow diagram of study selection and inclusion.

A total of 23 studies were deemed eligible for inclusion in this systematic review [16,17,23-44] (Figure 1); Tables 2 and 3 present the study characteristics. Sufficient data was available to allow analysis of just one variable: grams per week of ethanol consumed. If sufficient data to calculate means and SDs for this outcome were not reported in the published article, authors were contacted. Data were provided by the authors for 3 studies [17,39,41]. Data on alcohol consumption that could be transformed into grams per week of ethanol were not collected in 2 studies [25,27] and insufficient data to calculate the weighted mean difference (WMD) in grams of ethanol per week were reported in 4 studies [23,28,29,42]. Therefore, a total of 17 studies were included in the meta-analysis (1 study was published in 2 papers [16,17]. Most of these studies were conducted with student populations (13/17, 76%) and in the United States (10/17, 59%). All study interventions were either computer- or Web-based. The content of the interventions included an assessment followed by personalized and/or normative feedback. Control conditions generally consisted of an assessment with no further feedback, but 4 studies included general information on alcohol consumption for those in the control conditions [25,28,33,35]. There was some variation in the dose of the intervention with the reported time taken to complete the intervention ranging from less than 5 minutes [34] to 45 minutes [37]. The dose of exposure to the intervention could also be increased through repeated access during the study period [24] and/or a printed copy of the personalized feedback provided [26,31,36,38,40,43]. The attrition rate was highly variable between studies, ranging from 1% or 2% (eg, Hester et al [30]) up to more than 50% [42]

**Table 2:** Size and nature of study population and method of recruitment.

Study ID^a^		Male, n (%)	Mean age (SD)	Population	Recruitment
**Araki et al, 2006 [23]**		24 (100)		Japan, employees of a manufacturing plant with available annual health check-up data	Not reported
	eSBI (n=12)		44.3 (7.2)		
	Control (n=12)		43.8 (7.3)		
**Blankers et al, 2011 [24]**				Netherlands, adult general population	Visitors to the Collaborating Substance Abuse Treatment (SATC) website
	eSBI (n=68)	40 (58.8)	41.1 (9.6)		
	Control (n=69)	35 (50.7)	43.7 (9.3)		
**Boon et al, 2011 [25]**		450 (100)		Netherlands, adults in the general population	Nationally representative online household survey
	eSBI (n=230)		40.6 (15.2)		
	Control (n=220)		40.3 (15.1)		
**Butler et al, 2009 [26]**				United States, undergraduate university students	Not reported
	eSBI (n=30)	11 (36.7)	20.6 (1.48)		
	Control (n=26)	9 (34.6)	20.4 (1.49)		
**Cunningham et al, 2009 [16]; Cunningham et al, 2010 [17]**				Canada, adults in the general population	Randomly selected from an on-going general population telephone survey
	eSBI (n=92)	53 (57.6)	39.5 (13.5)		
	Control (n=93)	45 (48.4)	40.8 (13.4)		
**Cunningham et al, 2012 [27]**		118 (52.5)	22.6 (12.2)	Canada, university students	Randomly selected using student email addresses
	eSBI (n=211)				
	Control (n=214)				
**Ekman et al, 2011 [28]**				Sweden, third-year university students	Email invitation to all third-year students
	eSBI (n=330)	152 (46.1)	N (%):18-20=43 (13), 21-25=264 (80), ≥26=23 (7)		
	Control (n=324)	120 (37.0)	N (%) : 18-20=49 (15), 21-25=233 (72), ≥26=29 (9)		
**Hansen et al, 2012 [29]**				Denmark, adults in the general population	Identified through the Danish Health Examination Survey, those identified as heavy drinkers were sent an email invitation to take part
	eSBI PFI (n=476)	271 (56.9)	median=61		
	eSBI PBA (n=450)	246 (54.7)	median=59		
	Control (n=454)	244 (53.7)	median=60		
**Hester et al, 2012 [30]**				United States, university students	Identified through advertisements in the college newspaper and around the campus
	Exp 1: eSBI (n=65)	41 (63.1)	20.5 (1.80)		
	Exp 1: Control (n=79)	49 (62.0)	20.3 (1.63)		
	Exp 2: eSBI (n=42)	23 (54.8)	20.0 (1.52)		
	Exp 2: Control (n=40)	23 (57.5)	20.3 (2.09)		
**Hester et al, 2005 [31]**		32 (52.5)	Males=46.1 (13.8); females=45.2 (9.4)	United States, adult general population	Identified through advertisements in the media
	eSBI (n=35)				
	Control (n=26)				
**Kypri et al, 2009 [32]**				Australia, random sample of undergraduate university students	Students were sent a letter by mail followed by an email containing a Web link to the study questionnaire; up to 4 email reminders were sent
	eSBI=1251	687 (54.9)	19.7 (1.8)		
	Control=1184	645 (54.5)	19.7 (1.8)		
**Kypri et al, 2008 [33]**				New Zealand, users of a university student health service	Those leaving the student health service reception desk were consecutively approached and invited to participate
	eSBI (n=138)	67 (48.6)	20.1		
	Control (n=146)	70 (47.9)	20.1		
**Kypri et al, 2013 [34]**				New Zealand, Maori university students	Invited by email with up to 3 reminder emails
	eSBI (n=939)	35.7	20.2 (1.9)		
	Control (n=850)	33.2	20.1 (2.2)		
**Kypri et al, 2004 [35]**		52 (50.0)		New Zealand, users of a university student health service	Those checking into the reception of the student health service were invited to take part
	eSBI (n=51)		19.9 (1.4)		
	Control (n=53)		20.5 (1.8)		
**Lewis et al, 2007 [36]**			18.5 (2.04)	United States, university students enrolled in first-year orientation	All students enrolled for first-year orientation were invited to take part
	eSBI specific (n=75)				
	eSBI neutral (n=82)				
	Control (n=88)				
**Murphy et al, 2010 [37]**			18.6 (1.2)	United States, university students	Students enrolled in introductory classes were invited to take part
	eSBI (n=45)				
	Control (n=42)				
**Neighbors et al, 2004 [38]**		104 (41.3)	18.5 (1.24)	United States, university students from psychology classes	Students attending psychology classes were invited to take part
	eSBI (n=126)				
	Control (n=126)				
**Neighbors et al, 2010 [39]**		208 (42.4)		United States, incoming university freshmen students	Incoming university freshmen were invited to complete a Web-based survey sent via email and post
	eSBI GSF (n=163)				
	eSBI GNSF(n=164)				
	Control (n=163)				
**Neumann et al, 2006 [40]**				Germany, trauma center	Patients attending a trauma center were invited to take part after provision of initial care and resolution of significant pain
	eSBI (n=561)	449 (80.0)	median=30		
	Control (n=575)	449 (78.1)	median=31		
**Palfai et al, 2011 [41]**			18.6 (1.45)	United States, university students attending an introductory psychology class	Students attending an introductory psychology class were invited to take part
	eSBI (n=56)				
	Control (n=63)				
**Spijkerman et al, 2010 [42]**				Netherlands, volunteer members of an open access panel aged 15-20	Registered members of an open access panel were invited to take part via email
	eSBI NNF (n=192)	74 (38.5)	18.2 (1.55)		
	eSBI NF (n=193)	82 (42.5)	18.1 (1.54)		
	Control=190	69 (36.3)	18.1 (1.59)		
**Wagener et al, 2012 [43]**				United States, university students	Invited to participate via email using an online participant pool management system
	eSBI (n=39)	18 (46.2)	20.3 (1.67)		
	Control (n=37)	19 (51.4)	20.3 (1.49)		
**Walters et al, 2009 [44]**			19.8 (SD not reported)	United States, university students	University students invited via email, presentations, and posters at the university
	eSBI (n=67)				
	Control (n=69)				

^a^ eSBI: electronic screening and brief intervention; GNSF: gender-nonspecific; GSF: gender-specific; NF: intervention with normative feedback; NNF: intervention without normative feedback; PBA=personalized brief advice intervention; PFI=brief personalized feedback intervention.

**Table 3:** Characteristics of screening, experimental, and control interventions, and nature and timing of assessments.

Study ID	Screening cutoff^a^	eSBI details^a^	Control group	Dropouts at follow-up, n (%)^a^
Araki et al, 2006 [23]	Abnormal levels of gamma-glutamyl transpeptidase	Personalized feedback and advice sent via 2 emails 1 month apart; encouraged to ask questions via email	Assessment only	2 mo: (1 participant was not included in the analysis but the group that they were randomized to was not reported)
Blankers et al, 2011 [24]	AUDIT score ≥8 and reported drinking average 14 standard drinks per week	Access to an online self-help program based on motivational interviewing and cognitive behavioral therapy principles, suggested daily use for 4 weeks	Assessment only	3 mo: eSBI: 20 (29.4), control: 18 (26.1)
Boon et al, 2011 [25]	Exceeding Dutch guideline for low risk drinkers (>20 alcohol units per week or > 5 alcohol units on a single occasion on at least 1 day/week)	Single, 20-min brief personalized feedback session through website with the opportunity to print the feedback	Assessment and educational leaflet, instructed to read the leaflet for 20 min and could print the material	1 mo: eSBI: 18 (7.8), control: 19 (8.6) 6 mo: eSBI: 22 (9.6), control: 25 (11.4)
Butler et al, 2009 [26]	≥2 binge drinking occasions (≥5 drinks in 1 sitting for men and 4 or more for women) and 2 alcohol-related problems in the past 28 days Standard drink=14 g ethanol	Single, average 11-min session of computer-delivered personalized feedback and a paper copy to take home	Assessment only	4 w: eSBI: 9 (30.0), control: 4 (15.4)
Cunningham et al, 2009 [16] and Cunningham et al, 2010 [17]	Score ≥4 on the AUDIT-C (standard drink=13.6 g ethanol)	Single, 10-min session completing Check Your Drinking online intervention of normative and personalized feedback	Assessment and a list of possible components to include in an intervention	3 mo: eSBI=7 (7.6), control: 3 (3.2) 6 mo: eSBI: 7 (7.6), control: 8 (8.6) 12 mo: eSBI: 11 (12.0), control: 11 (11.8)
Cunningham et al, 2012 [27]	Score ≥4 on the AUDIT-C	Access to the Check Your Drinking University version online intervention of normative and personalized feedback; intervention could be accessed repeatedly	Assessment only	6 w: eSBI: 59 (28.0), control: 75 (35.0)
Ekman et al, 2011 [28]	(1) Weekly alcohol consumption >120 g ethanol (women) or 180 g ethanol (men) in a typical week in the past 3 months and/or (2) engaged with heavy episodic drinking defined as consuming ≥48 g of ethanol (women) or ≥60 g of ethanol (men) on ≥2 occasions in the past month	Single session intervention of personalized normative feedback delivered via email	Assessment and brief feedback consisting of 3 statements	3 mo: eSBI: 125 (37.9), control: 113 (34.9) 6 mo: eSBI: 78 (24), control: 80 (24)
Hansen et al, 2012 [29]	Above recommended max drinking limit set by the Danish National Board of Health of 14 drinks/168 g ethanol for women or 21 drinks/252 g for men (standard drink=12 g ethanol)	PFI: fully automated, Internet-based single session of brief personalized and normative feedback; PBA: fully automated, Internet-based single session of brief personalized feedback and advice	Assessment only	6 mo: eSBI PFI: 186 (39.0), eSBI PBA: 171 (38.0), control: 150 (33.0) 12 mo: eSBI PFI: 109 (22.9), eSBI PBA: 108 (24.0), control: 95 (20.9)
Hester et al, 2012 [30]	Met the National Institute for Alcohol and Alcohol Abuse (2004) criteria for heavy episodic drinking of ≥4 drinks per occasion (women) or ≥5 drinks per occasion (men) at least once in past 2 weeks and an estimated peak blood alcohol concentration of 80 mg% or more (standard drink=14 g ethanol)	Self-guided College Drinkers Check-up, delivered online, single session taking up to 35 min to complete; assessment, normative feedback, and advice	Assessment only	Exp 1 (1 mo): eSBI: 2 (3.1), control: 2.5) Exp 1 (12 mo): eSBI: 6 (9.2), control: 8 (10.1) Exp 2 (1 mo): eSBI: 0 (0.0), control: 1 (2.5)
Hester et al, 2005 [31]	Score ≥8 AUDIT (standard drink=14 g ethanol)	Computer-based DCU, assessment, feedback, and decision-making modules; single session can take up to 90 min to complete with the option of printing the feedback	Assessment only	4 w: not reported
Kypri et al, 2009 [32]	Score ≥8 on AUDIT and exceeding the Australian National Health and Medical Research Councils guideline for acute risk (defined as 4 standard drinks for women or 6 for men in a single occasion in the last 4 weeks); standard drink=10 g ethanol	Single online session of personalized feedback	Assessment only	1 mo: eSBI: 288 (23.0), control: 237 (20.0) 6 mo: eSBI: 442 (35.3), control: 420 (35.5)
Kypri et al, 2008 [33]	AUDIT score ≥8; standard drink=10 g ethanol	Single computer-delivered session of personalized and normative feedback taking a median 9.3 min to complete	Assessment and alcohol facts leaflet	6 mo: eSBI: 22 (15.9), control: 22 (15.1) 12 mo: eSBI: 25 (18.1), control: 20 (13.7)
Kypri et al, 2013 [34]	Score ≥4 on AUDIT; standard drink=10 g ethanol	Single online session of personalized and normative feedback taking a median 4.3 min to complete	Assessment only	5 mo: eSBI: 207 (22.0), control: 170 (20.0)
Kypri et al, 2004 [35]	Score ≥8 on AUDIT and consuming >4 standard drinks for men or >6 for women on ≥1 occasion in past 4 weeks (standard drink=10 g ethanol)	Computer-delivered single session of personalized feedback	Assessment and alcohol facts leaflet	6 w: eSBI: 9 (17.6), control: 12 (22.6) 6 mo: eSBI: 4 (7.8), control: 6 (11.3)
Lewis et al, 2007 [36]	≥1 heavy episode (≥4 standard drinks in 1 sitting for women and ≥5 standard drinks in 1 sitting for men) in the previous month; standard drink=14 g ethanol	eSBI specific: gender-specific Web-based personalized normative feedback; eSBI neutral: gender-neutral Web-based personalized normative feedback; feedback was read on screen and participants were given printout to take home	Assessment only	5 mo: eSBI specific: 11 (14.7), eSBI neutral: 15 (18.3), control: 10 (11.4)
Murphy et al, 2010 [37]	≥2 heavy drinking episodes in the past month (described as ≥4 standard drinks on 1 occasion for women and ≥5 standard drinks for men) or ≥1 heavy drinking episodes for minority students; standard drink=14 g ethanol	Interactive, Web-based intervention, E-CHUG (Electronic Check-up and Go), assessment and personalized feedback in a single session lasting up to 45 min with a brief comprehension test on completion	Assessment only	1 mo: eSBI: 7 (15.6), control: 3 (7.1)
Neighbors et al, 2004 [38]	≥1 heavy drinking episode in the previous month (defined as 4 standard drinks in 1 sitting for women and 5 standard drinks for men); standard drink=14 g ethanol	Single computer-delivered session of personalized normative feedback presented on screen for 1 min plus a printout	Assessment only	3 mo: whole sample: 53 (21.0) 6 mo: whole sample: 45 (17.9)
Neighbors et al, 2010 [39]	≥5 drinks for men and ≥4 drinks for women on ≥1 occasions in the past month; standard drink=14 g ethanol	eSBI GSF: single session delivered online giving personalized gender-specific feedback; eSBI GNSF: single session delivered online giving personalized gender-nonspecific feedback	Assessment and an attention test (facts about the university students were presented in the same format as the intervention)	6 mo: eSBI GSF: 10 (6.1), eSBI GNSF: 16 (9.8), control: 13 (8.0) 24 mo: eSBI GSF: 33 (20.2), eSBI GNSF: 25 (15.2), control: 31 (19.0)
Neumann et al, 2006 [40]	AUDIT score ≥5	Single session of computer-generated feedback and a printout to take home	Assessment only	6 mo: eSBI:=213 (37.9), control: 207 (36.0) 12 mo: eSBI: 252 (44.9), control: 224 (39.0)
Palfai et al, 2011 [41]	Hazardous drinkers who either (1) consumed alcohol in the past month and scored ≥8 on AUDIT or (2) reported ≥2 heavy drinking episodes (defined as ≥5 drinks for men or ≥4 drinks for women in the past month; standard drink=14 g ethanol)	Single computer-delivered session of personalized, normative, and gender-specific feedback	Assessment and health guidelines for sleep and consumption of fruit and vegetables	1 mo: whole sample: 0 (0.0)
Spijkerman et al, 2010 [42]	Age 15-16 y: engage in binge drinking at least once a month; age 17-20 y: engaged in binge drinking ≥1/week; binge drinking defined as drinking ≥4 alcoholic drinks for women or ≥6 for men on 1 occasion; standard drink=10 g ethanol	eSBI NNF: single online session of personalized feedback tailored to age and gender, took ~15 min to complete; eSBI NF: single online session of personalized normative gender- and age-specific feedback, took ~15 min to complete	Assessment only	1 mo: eSBI NNF: 92 (47.9), eSBI NF: 93 (48.2), control: 68 (35.8)) 3 mo: eSBI NNF: 106 (55.2), eSBI NF: 104 (53.9), control: 87 (45.8)
Wagener et al, 2012 [43]	≥1 heavy drinking session (≥5 drinks on 1 occasion for men or ≥4 for women), drinking ≥20 drinks/month on average and experiencing negative consequences of that use in the last month (standard drink=14 g ethanol)	Single session using of computer-delivered assessment personalized feedback using an interactive program (DRAFT-CS), took ~45 min to complete; participants were given printout of their feedback	Assessment only	10 w: eSBI: 2 (5.1), control: 3 (8.1)
Walters et al, 2009 [44]	Reported ≥1 heavy drinking session in the past 2 weeks defined as ≥5 standard drinks for men and ≥4 standard drinks for women (standard drink=14 g ethanol)	eSBI: single session of personalized feedback delivered through the online Check-Up to Go	Assessment only	3 mo: eSBI: 9 (13.4), control: 6 (8.7) 6 mo: eSBI: 13 (19.4), control: 8 (11.6)

^a^ eSBI: electronic screening and brief intervention, AUDIT: Alcohol Use Disorders Identification Test, PFI: brief personalized feedback intervention, PBA: personalized brief advice intervention, DCU: Drinkers Check Up, GSF: gender-specific, GNSF: gender-nonspecific, NNF: intervention without normative feedback, NF: intervention with normative feedback, FBO: feedback only, DRAFT-CS: Drinking Assessment and Feedback Tool for College Students.

### Grams of Ethanol per Week

**Figure 2:**
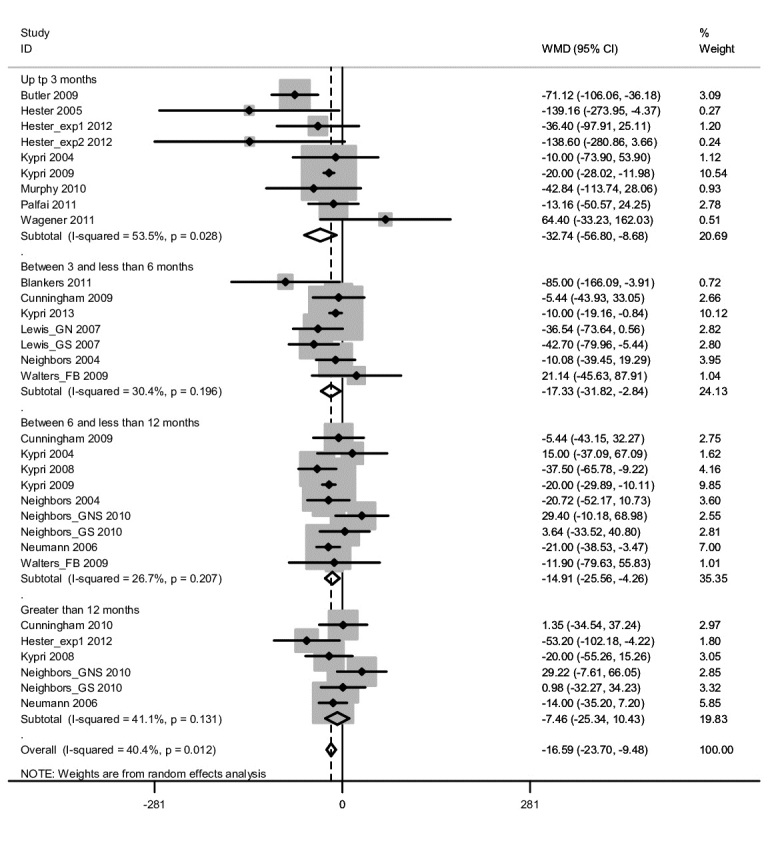
Forest plot for weighted mean difference (WMD) in grams of ethanol per week at follow-up between those in the eSBI group and controls.

**Figure 3:**
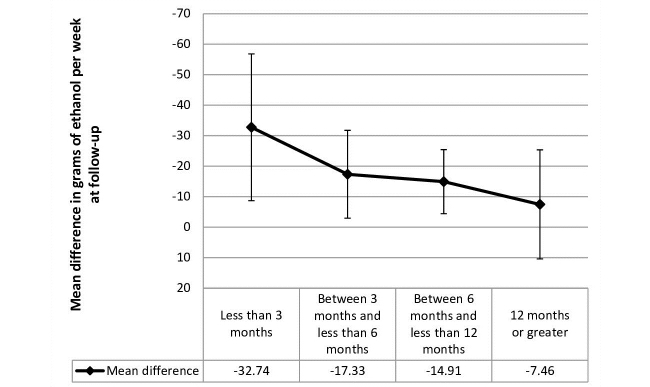
Mean difference in grams of ethanol per week at follow-up postintervention with 95% confidence intervals.

Nine studies included data for a follow-up period of up to 3 months (mean 1.06 months, SD 0.18), 6 studies with a follow-up period between 3 and less than 6 months (mean 3.86 months, SD 1.07), 8 studies with a follow-up period between 6 and less than 12 months (all included studies had a follow-up period of 6 months ), and 5 studies included data for a follow-up period greater than 12 months (mean 16 months, SD 6.20) (Figure 2). There was a statistically significant difference in pooled mean difference in grams of ethanol per week consumed between those who received the eSBI and controls for follow-up period subgroups up to 3 months, between 3 and less than 6 months, and between 6 and less than 12 months (Table 4). This difference represents a significantly lower mean number of grams of ethanol consumed per week at follow-up by those in the eSBI group compared to controls. There was no statistically significant difference between groups in pooled mean difference in grams of ethanol per week for long-term follow-up. The greatest difference was found at less than 3 months follow-up, which decreased with length of follow-up (Figure 3).

There was statistically significant and moderate heterogeneity between studies included at less than 3 months follow-up. Heterogeneity was not statistically significant for any of the other follow-up groups. Egger’s test was not statistically significant for all follow-up periods, indicating an absence of publication bias.

**Table 4:** Results of meta-analysis including significance test and heterogeneity statistics.

Follow-up period	Sample size, n		Mean difference significance test		Heterogeneity statistic			
	Experimental	Control	*z*	*P*	*Q*	*df*	*P*	I^2^
<3 months	1305	1307	2.67	.01	17.19	8	.03	53.5%
3-6 months	1211	811	2.34	.02	8.62	6	.20	30.4%
6-12 months	1921	1751	2.74	.01	10.91	8	.21	26.7%
≥12 months	899	816	0.82	.41	8.49	5	.13	41.1%

### Sensitivity Analysis

Participants in the intervention arm of the study conducted by Blankers et al [34] had access to the online self-help intervention at any time, but it was suggested that they access it daily during a 4-week period. This methodology is different from other studies included in this review in that the other studies allowed participants access to the electronic intervention for a single session. Therefore, sensitivity analysis was conducted to assess the impact of this study on the overall mean difference in consumption of alcohol between 3 and less than 6 months. Removal of the Blankers et al [34] study from the meta-analysis had little effect on the mean difference in grams of alcohol consumed per week for those in the intervention groups compared to controls (mean difference -13.40, 95% CI -23.94 to 2.85).

The length of the intervention in the study conducted by Hester et al [31] was on average 90 minutes; this is longer than the definition of brief intervention for eligibility of inclusion in this systematic review and meta-analysis. However, because the intervention was completed in 1 session it was decided that a sensitivity analysis would be conducted to explore the impact of this study on the pooled mean difference in alcohol consumption at up to 3 months. Removal of the study conducted by Hester et al [31] had minimal impact on the pooled mean difference in grams of alcohol consumed per week for those in the intervention group compared to controls (mean difference -29.53, 95% CI -52.50 to 6.56).

### Risk of Bias

The quality of the evidence reviewed was considered to be acceptable with most studies included in this review assessed as being adequate in terms of their methodological quality. Three studies were considered to be of high methodological quality [24,32,34]. The addition of future research may have an impact on the conclusions of the review and meta-analysis.

## Discussion

The results of this systematic review and meta-analysis suggest that eSBI is effective in reducing alcohol consumption in the follow-up postintervention period of less than 3 months, between 3 months and less than 6 months, and between 6 months and less than 12 months, but not in the longer term follow-up period of 12 months or longer. The overall mean difference in grams of ethanol per week consumed between those in the intervention and controls groups was 16.59 (Figure 2), which is equivalent to 2 standard drinks in the United Kingdom (1 standard drink=8 g ethanol). This difference is somewhat smaller compared to a previous review, which found an overall mean difference of 25.88 g of ethanol per week [14]. The current review did not include studies of treatment seeking populations or those in which individuals were randomized regardless of their drinking status at baseline; this may account for some of the variation in mean difference in alcohol consumption. Furthermore, there may have been a variation in the length of follow-up for studies included in the current research and Khadjesari et al’s [14] meta-analysis. The inclusion of more studies with a shorter follow-up length may have resulted in an inflated overall mean difference in alcohol consumption between controls and those who received the intervention.

The pattern of results found here are in-line with the results of Cunningham et al [16,17]. They reported significantly lower levels of weekly alcohol consumption in those who received a Web-based brief intervention compared to controls at 3 and 6 months, but not at 12-month follow-up. Cunningham et al [16,17] is the only eSBI study included in this systematic review and meta-analysis to follow up participants over the 3 time points: 3-, 6-, and 12-month follow-ups. Meta-analysis allowed for replication of their results with a much larger sample size. The magnitude of the effect in this study reduced with increasing length of the follow-up period, from nearly 4 standard drinks at a follow-up point of less than 3 months to less than 1 standard drink at a longer duration of follow-up of 12 months or greater, indicating a decline in the effectiveness of eSBI to significantly reduce alcohol consumption. All the data included in this review were from studies using a single eSBI session, although the option of returning to the eSBI was available for one study [23] and a printout of personalized feedback was generally offered (see Table 3). Neighbors et al [39] found no compelling evidence to suggest that multiple doses of electronic personalized brief advice, administered every 6 months for 2 years, was more effective than a single one-off intervention.

There was a variation in the extent of eSBI delivered between studies included in this review with some interventions taking substantially longer to complete and one study encouraged daily use of their online self-help program [24]. It is possible that more extensive interventions will have a greater impact on alcohol consumption. However, a recent large cluster randomized controlled study of face-to-face SBI in primary care found no difference in effectiveness between an information leaflet, 5 minutes of structured brief advice, or 20 minutes of brief lifestyle counseling on proportion of individuals with a negative AUDIT score (<8) at 6- and 12-month follow-ups [5]. Furthermore, a meta-analysis of face-to-face SBI found that although the reduction in alcohol consumption (grams of ethanol per week) was greater for more substantial interventions (including those that were longer in duration and administered in more than one session) compared to less intensive interventions, the difference was not statistically significant [4]. To date there has been no comparable studies for eSBI.

A large attrition rate (up to 55%) has been noted in some of the eSBI studies included in this review. High attrition rates are common in electronic interventions for nontreatment-seeking individuals and reasons for this are likely to be complex and varied [45]. Attrition will have an obvious impact on the validity of results obtained and introduce bias, for example, those more committed to reducing their alcohol intake may remain in the trial and inflate positive alcohol outcomes. This has led to research into ways of reducing attrition using incentives. Khadjesari et al [46] investigated whether attrition could be improved in their study of a Web-based intervention (Down Your Drink) for reducing alcohol consumption by incentivizing study completion. Participants were randomized to receive no incentive, a £5 Amazon voucher, £5 donation to Cancer Research, or entry into a £250 prize draw. There was no significant difference in response rate between any of the study arms. A second study by Khadjesari et al [46] randomized participants to receive a higher value incentive of £10 Amazon voucher or no incentive. This resulted in a 9% difference in response rate between the 2 groups, suggesting that appropriate incentivization can reduce participant attrition. However, some caution is required when considering the use of incentives to reduce attrition in online interventions. In the previous study, incentives were given on completion of the intervention and follow-up, rather than on sign-up to the intervention; this prevented individuals signing up who were only doing so for the incentive not the potential benefits of the research. Further exploration of the mechanism of action of incentives is required in eSBI, socioeconomic status, cultural factors, and reasons for attrition may all influence how effective incentives are at improving attrition in research [45].

Seventeen studies were included in the meta-analysis and most of these took place in the United States and with student populations. Binge drinking among young adult and student populations continues to be a concern. In the United Kingdom, 45% of males and 46% of females aged 16-24 years drink more than twice the recommended amount of alcohol (3-4 units for males and 2-3 units for females) in a single session in the previous week [47]. Binge drinking can increase the risk of behaviors that are illegal, violent, or risky (eg, unprotected sex) [48,30,48]. Binge drinking at university may also lead to long-term problems with physical and mental health [48]. This may help to explain why the majority of studies included in this systematic review and meta-analysis were conducted with student populations. Furthermore, the population of a university is generally large with up-to-date information technology facilities, which would be ideal for the implementation of an eSBI. A culture of binge drinking is evident among student populations; the pattern of drinking is likely to be somewhat different to the general population. Because of the limited number of relevant eSBI trials available, further analysis to investigate the impact of population on the effectiveness of eSBI is needed. Therefore, the generalizability of the current findings for the general population is not known.

The studies included in this meta-analysis also varied in the length, content, and theoretical basis of the intervention. Although almost all the included studies incorporated an element of personalized feedback as part of the intervention, there remains variation in both the mechanism and the context of how this was delivered. Further investigation into the effective components of these interventions was not possible and this should form an area for future research.

A comprehensive search strategy was used to identify relevant published randomized controlled trials for inclusion in this review and meta-analysis. However, it is possible that some trials may have been missed because unpublished research was not sought although an Egger’s test suggested that no publication bias was present.

The results of this systematic review and meta-analysis demonstrate significant reductions in weekly alcohol consumption between intervention and control conditions at a follow-up point of less than 3 months, between 3 and 6 months, and between 6 and 12 months; as such, eSBI should be judged an effective intervention, a recent review of effective interventions targeting adolescent populations adds further support for the use of Web-/smartphone-based technology [49]. Advantages inherent to eSBI, such as reduced cost of implementation and wider accessibility compared to conventional face-to-face SBI, should also be considered. However, because of a lack of consistency in reporting of alcohol consumption outcome measures, this review could only report on grams of ethanol consumed per week. A greater consensus in the reporting of outcome measures and more uniform reporting of the content and theoretical basis of eSBI would result in the ability to make more robust conclusions regarding the effectiveness of eSBI in reducing alcohol consumption and alcohol-related harms in the longer term.

## Abbreviations

AUDIT: Alcohol Use Disorders Identification Test
eSBI: electronic screening and brief intervention
MCV: mean corpuscular volume
NIHR: National Institute for Health Research
SBI: screening and brief intervention
SIGN: Scottish Intercollegiate Guidelines Network
